# Assessing psychological variables on mobile devices: an introduction to the experience sampling app *ESM-Quest*

**DOI:** 10.3389/fpsyg.2023.1271422

**Published:** 2024-01-26

**Authors:** Thomas Goetz, Wolfgang Steiner, Elisabeth Graf, Lisa Stempfer, Christina Ristl, Fiona S. Rupprecht, Johanna L. Donath, Elouise Botes, Jana Nikitin

**Affiliations:** ^1^Department of Developmental and Educational Psychology, Faculty of Psychology, University of Vienna, Vienna, Austria; ^2^Cognitive Science and Assessment, Faculty of Humanities, Education, and Social Sciences, University of Luxembourg, Luxembourg, Luxembourg

**Keywords:** experience sampling method, *ESM-Quest*, psychological variables, real-time data, state, trait, assessment

## Abstract

The use of the Experience Sampling Method (ESM), which involves repeated assessments in people’s daily lives, has increased in popularity in psychology and associated disciplines in recent years. A rather challenging aspect of ESM is its technical implementation. In this paper, after briefly introducing the history of ESM and the main reasons for its current popularity, we outline the *ESM-Quest* experience sampling app which is currently being developed at the University of Vienna. *ESM-Quest* runs on different operating systems, specifically on mobile devices such as smartphones and tablets running either iOS or Android. An internet connection is not necessary during the assessment. Compared to most other ESM apps, *ESM-Quest* allows event-based random sampling, which is very helpful when assessments need to be collected within specific situations. Currently, *ESM-Quest* is being utilized at the University of Vienna and will be made available for research groups worldwide upon request. We introduce the technical aspects of *ESM-Quest* and provide examples of analyses on ESM data collected through this app, such as examining fluctuations in constructs within individuals. Finally, we outline potential next steps in ESM research.

## Introduction

Experience sampling method (ESM), also referred to as “ecological momentary assessment” and “ambulatory assessment” (see Trull and Ebner-Priemer, 2014), is a method of data collection. Sampling procedures gather self-report data repeatedly during real-world experiences to provide real-time data (i.e., *in situ* self-reports), as opposed to retrospective reports, on individuals’ perceptions (Csikszentmihalyi and Larson, 1987). Experience sampling has a long tradition in research. Diary studies, which can be seen as predecessors or even simple forms of experience sampling, were already used in the early 1900s (Bevans, 1913). While only relatively few researchers used ESM until the 2000s, there has been a strong increase in studies using this method in the last 20 years (Wrzus and Neubauer, 2023). Currently, ESM is intensively used in research on mood disorders and dysregulation (Ebner-Priemer and Trull, 2009), substance usage (Shiffman, 2009), binge eating (Haedt-Matt and Keel, 2011), human-computer interaction (Consolvo and Walker, 2003), and in organizational research (Beal, 2015).

Several reasons might have contributed to the increase of ESM studies across disciplines, including psychology. Firstly, ESM data demonstrate *high ecological validity* as they reflect a person’s experiences in the “here and now,” during their interaction with their natural environment. Thus, retrospective memory and reporting biases (e.g., “Rosy retrospection”; Mitchell et al., 1997) are assumed to play a more minor role in ESM assessments in comparison to traditional trait questionnaire-based research. Furthermore, stereotypes that might impact responses to traditional trait questionnaires are assumed to have only a limited impact on ESM data, where answers to the questions are typically more spontaneous and thus less prone to stereotypes (Goetz et al., 2013). Secondly, ESM allows for the *investigation of within-person processes*, also known as interindividual analyses or “idiographic research” (see Lamiell, 1997). This type of investigation is currently rather prominent because analyzing processes within individuals strongly contributes to our understanding of human psychological mechanisms by going beyond findings based on interindividual data (Murayama et al., 2017). Thirdly, ESM can *contribute to the call for the use of multiple methodologies to study constructs*, such as combining ESM with retrospective questionnaires, facial recognition, heart rate monitoring, etc. Fourthly, real-time assessments are a *prerequisite for the use of adaptive technical learning systems*. The quality of adaptive systems strongly depends on the quality of the assessment of an individual’s current state. Fifthly, there has been significant *development in statistical methods for analyzing intraindividual data* in the last 20 years (see Gabriel et al., 2019). This allows for the strong utilization of the advantages of ESM data from a statistical perspective. Finally, although ESM was once a time- and cost-intensive method of data assessment, technological advancements in the past 20 years have made ESM *relatively easy to implement* (Doherty et al., 2020).

With respect to ESM as a specific type of data assessment, several articles exist that provide an overview of core topics related to this method (e.g., Goetz et al., 2016a; Van Berkel et al., 2017; Gabriel et al., 2019; Doherty et al., 2020; Wrzus and Neubauer, 2023). Such topics include the choice of an appropriate sample size for participants (e.g., Van Berkel et al., 2017), the number of assessment time points within individuals (e.g., Doherty et al., 2020), the type of sampling (interval-contingent, event-contingent, signal-contingent sampling; Scollon et al., 2003), and the duration of the assessment period (e.g., spanning two weeks; e.g., Van Berkel et al., 2017). Other aspects include the selection of scales for ESM assessments (e.g., using single items; e.g., Goetz et al., 2016a), the use of ESM incentives (e.g., Wrzus and Neubauer, 2023), handling missing data (e.g., Silvia et al., 2013), the statistical methods employed to analyze ESM data (e.g., Goetz et al., 2016a), and the technical implementation of the project (e.g., software; for an overview, see Van Berkel et al., 2017). In addition, the combination of ESM assessments with other types of assessments, such as the measurement of physiological data or the use of video data has been an important advancement for the use of the method (e.g., Roos et al., 2023). In general, most of these aspects are interrelated, resulting in primarily individualized approaches to conducting ESM studies.

In terms of the technical implementation of ESM studies, various solutions are currently available. For instance, there are commercial apps, and different research groups have developed their own systems, such as apps, electronic pagers, and phone signals (Van Berkel et al., 2017). However, even though some of these systems are relatively user-friendly, most carry a prohibitive financial cost. Furthermore, many systems require an internet connection during the data assessment, which is at times not possible (e.g., assessments during leisure time, at schools). Furthermore, the vast majority of existing apps do not support event-based random sampling, which is essential for assessing data in specific situations. For example, conducting several randomized assessments within a math class at school (i.e., the event) would require an app that can be activated by students at the beginning of the math class.

In this paper, we introduce *ESM-Quest*, an experience sampling app developed at the Faculty of Psychology, University of Vienna (Austria). *ESM-Quest* can be utilized on mobile devices, such as smartphones and tablets, running on either iOS or Android. During data assessment, a connection to the internet is not necessary. A highly helpful feature of *ESM-Quest* is that it allows for event-based random sampling. The app has been tested and is currently in use at the University of Vienna, but will be made available to research groups worldwide upon request.

In this paper, we present the technical aspects of the app and provide examples of analyses conducted on ESM data collected using *ESM-Quest*. Further, we outline potential next steps in ESM research. One of the main objectives of this work is to motivate researchers to conduct ESM studies by demonstrating that ESM implementation is relatively straightforward and offers numerous opportunities to address research questions that are challenging or even impossible to explore using traditional trait assessments. In this regard, *ESM-Quest* offers a viable and convenient method for conducting ESM studies.

## Introduction of the experience sampling app *ESM-Quest*

### System overview

*ESM-Quest* consists of four main software components: the backend, the study administration frontend, an Android app, and an iOS app. The backend was developed with the open-source PHP framework Laravel and is hosted by the IT service provider of the University of Vienna. Its main task is to process, store, and provide data that is stored in a MySQL database, such as items, answers to those items, and study parameters. The backend provides a RESTAPI (Representational State Transfer Application Programming Interface) to receive data from and to send data to the other components. The study administration frontend application is based on the TypeScript open-source framework Angular and offers a user-friendly browser-based interface to manage *ESM-Quest* studies. The native mobile applications for data collection are written in Java (Android) and Swift (iOS).

### Main features

*ESM-Quest* enables researchers to conduct ESM studies in which participants can use their own mobile devices. Study participants can download the app for free from the Google Play Store or the Apple App Store. The configuration of the *ESM-Quest* study can be done in a browser-based web interface. While researchers at the University of Vienna use their single sign-on credentials to access the study administration, researchers not affiliated with the University of Vienna will be able to register for a guest account.

The system offers a questionnaire builder tool and different types of questionnaires including various answer formats (see below). A baseline questionnaire (e.g., demographic data, trait questionnaire) can be used at the beginning, a repeated state questionnaire during, and an end questionnaire (e.g., feedback) at the end of study participation.

Each *ESM-Quest* study defines a unique study code. Participants of the corresponding *ESM-Quest* study enter this code to participate (anonymously) in the study. All of the required content (e.g., texts and items) and parameters (e.g., number of signals) will be downloaded from the backend and stored on the participant’s device.

Study administrators must choose one of the following main variants of ESM studies: *random sampling* or *event-based random sampling*. While the *random sampling* mode leads to random signals during the entire period of study duration, the *event-based random sampling* mode requires that participants activate the system in predefined situations (e.g., at the beginning of a lesson at school) to get random signals within a configurable period of data collection. *Random sampling* mode requires the configuration of a timetable, where start and end time of random signals must be specified for each day of the week (multiple time intervals per day are currently not supported). In this mode the mobile device calculates all random points within the timeframe of the study under consideration of the study settings. In *event-based random sampling* mode, the timing of the signals is calculated each time the participant activates the system. The distribution of the random signals in both modes can be affected by setting the duration of the time interval of data collection, the number of random signals within the interval, and the minimum pause between two signals. The pause parameter helps to avoid clustering random signals.

Other modes, for example a pure event-based mode, where special events (e.g., physiological parameters or pressing buttons) trigger a state questionnaire are currently not available, but planned for future development.

For each calculated time point in both modes, an auditory signal is scheduled, at which point the smartphone sends a local notification to the participant with the request to fill out a state questionnaire. A clear strength of this approach is the possibility to run studies offline without an internet connection. In this case, a connection to the server is only mandatory while entering the study code and for sending answers to the server at the end of the study. If there is an internet connection on the mobile device during participation, the app tries to send all locally stored answers after each questionnaire to prevent data loss.

The collected data can be downloaded from the backend in CSV format or as a Microsoft Excel file in XSLX format. The data is divided into two parts. The first part contains the answers to each questionnaire. Answers in the dataset are linked to the timestamp of the signal for the corresponding state questionnaire. In the second part all these signal timestamps are listed together with the status of response for each questionnaire: *started* (questionnaire was opened) and *completed* (questionnaire was completed). Participants have no time limit to open a state questionnaire. The time between signal and starting a questionnaire can be calculated from the data. If a state questionnaire has not been answered before the next signal, it is marked as *missed* in the dataset.

In addition, for studies in *event-based random sampling* mode, the status *canceled* means that the planned questionnaires were cancelled due to a premature deactivation of the system by the user. In this mode, the time of activation and deactivation of the ESM system is listed for each timestamp.

### Study administration interface

**Figure 1:**
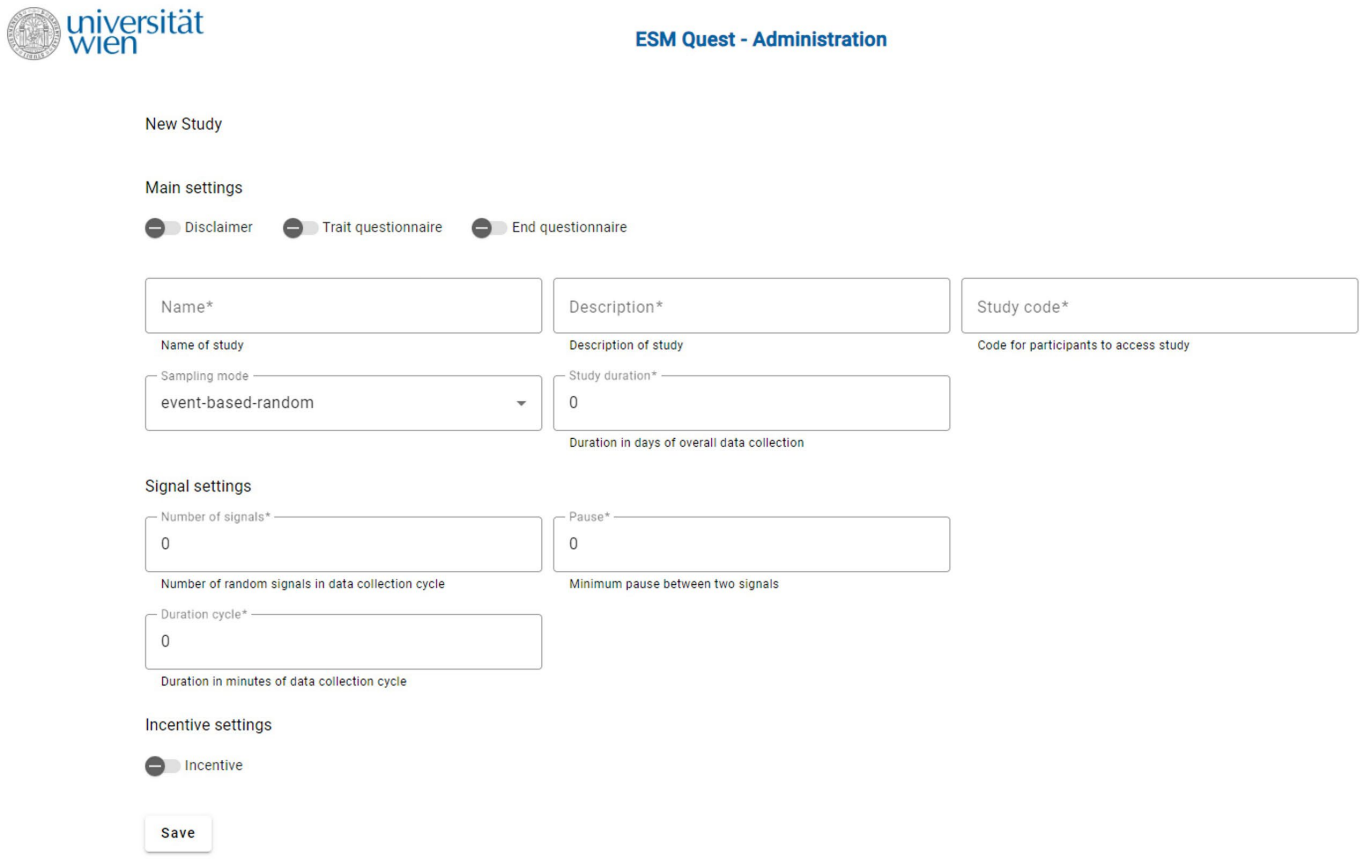
Screenshot of the ESM-Quest configuration options.

After entering the internet address for *ESM-Quest* study administration in the browser, a login screen is displayed. The next screen of study administration displays an overview of current studies of the logged-in user. The selection of a study or a click on the “New study” button leads to the first main menu point. In the “Settings” menu point the main settings of an *ESM-Quest* study can be made. Figure 1 shows a screenshot of the main configuration options.

Study administrators should first define the mode of the *ESM-Quest* study in the main settings: *random sampling* or *event-based random sampling*. The study duration field defines the days of overall data collection after entering the study code for participation on a mobile device. In this menu an optional disclaimer, the baseline questionnaire, and the end questionnaire can be activated. In the next main menu point of “Questionnaires,” a questionnaire builder tool is offered to create questionnaires. This tool supports different types of items and questionnaire content: Information texts, open-ended questions, single-choice items, multiple-choice items, Likert scales, and a slider question type. The main menu point “Texts” provides the possibility to customize most of the texts displayed in the app, for example, disclaimer text, instructions at the beginning, or acknowledgment messages at the end of the data collection. This customization also gives the possibility to create *ESM-Quest* studies in different languages. The last main menu point “Dashboard” is used for downloading the data and monitoring ESM studies in progress. The “Dashboard” displays an overview of the total response states overall as well as individually for each participant.

### Study participation

**Figure 2:**
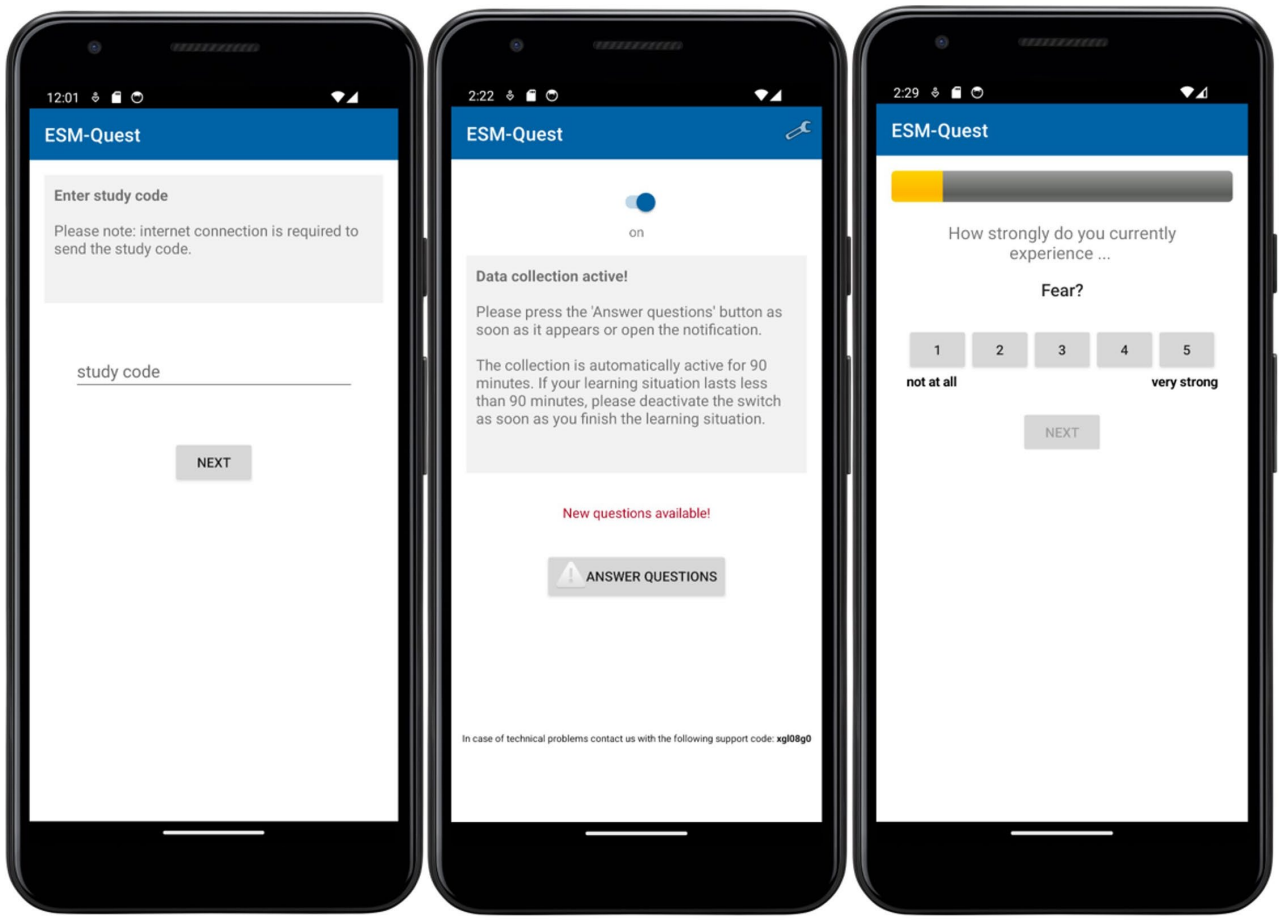
Screenshots of the mobile client of ESM-Quest.

After specifying all settings described above in the *ESM-Quest* study administration frontend, the study can be activated for testing or data collection. From this point, participants can take part in the *ESM-Quest* study with their Android or iOS devices. Figure 2 shows screenshots of the mobile client.

After downloading and installing, the *ESM-Quest* app asks for the study code. A valid study code results in downloading all corresponding settings, texts, and questionnaires to the smartphone. The next page shows an optional configurable disclaimer text that must be accepted with a checkbox to go further. If a baseline questionnaire has been configured in the *ESM-Quest* study settings, it will be displayed on the next pages. After finishing the baseline questionnaire, the app displays an information text in the *random sampling* mode, that the app can be closed now. In *event-based random sampling* mode, each time the *ESM-Quest* app is started, a screen with a slider button is shown, which activates the system for generating and sending signals.

Scheduled signals alert the user with a specific sound and vibration, depending on the device’s current alarm settings. Participants start a state questionnaire by clicking on the local notification or by opening the app. All items of the questionnaire are displayed side by side. After completing a questionnaire, the app can be closed again. When the period of data collection is over, the app optionally displays the end questionnaire, if this has been configured in the *ESM-Quest* study settings. On the last page of the app, there is a reminder to send all data to the server. After all data have been sent to the backend server, a configurable “thank you for participation”-message and an optional link for incentives are displayed. Participants are informed that the app can now be uninstalled.

More information about the app can be found on the website of *ESM-Quest*.^1^ Researchers who are interested in using the ESM-Quest App for their research projects can send a request to the email address provided on the ESM-Quest website.

## Examples for analyses of ESM data assessed via *ESM-Quest*

To illustrate the potential of *ESM-Quest*, we briefly present selected analyses of two studies in which this app was used. Both studies assessed perceived control, perceived value, and enjoyment in participants. Study 1 assessed these constructs in learning situations by using *event-based random sampling mode* in a student sample. Study 2 focused on adults in a lifespan sample using *random sampling*. To mirror the learning context of the student sample (Study 1) in the adult lifespan sample (Study 2), we only utilized a subsample of situations in which participants reported engaging in mental activities. Details about the study designs, sample descriptions, and items are summarized in Table 1.

**Table 1:** Study description of Study 1 and Study 2.

	Student sample (Study 1)	Adult lifespan sample (Study 2)
*Study design*		
Objective	Perceived control, perceived value, and enjoyment in different learning contexts at university	Everyday perceived control, perceived value, and enjoyment across random situations of daily life
Target group and situation	University students in learning situations	Adults participating in mental activities in their daily life
Baseline questionnaire (= Trait measures)	Demographics, single-item trait measures (wording parallel to state items)	Demographics, multi-item trait measures (not used for present analyses)
ESM sampling strategy (= State measures)	Event-based random sampling: Self-directed activation of the app in every learning situation, then 5 random signals for each activation period (minimum interval between signals: 10 min) over two weeks	Random sampling: 6 random signals (between 8 am and 8 pm) for a duration of 7 days
Data collection period	December 2022	October 2021 – January 2022
*Sample*		
Sample size	*N* = 97; 1,881 observations	*N* = 106; 729 observations
Age	18 to 31 years (*M* = 21.86, *SD* = 2.67)	18 to 85 years (*M* = 38.47, *SD* = 16.81)
Gender	82% female, 18% not available/missing data	68% female
State measures*		
Control	I feel in control of the current learning situation. *Not true at all* (1) to *Completely true* (5)	In this situation, I felt capable of what I was doing. *Does not apply at all* (1) to *Applies completely* (5)
Value	Understanding the current content is of great personal importance to me. *Not true at all* (1) to *Completely true* (5)	How did you experience your activity? *Not at all important for me personally* (1) to *Very important for me personally* (5)
Enjoyment	How strongly are you experiencing enjoyment at the moment? *Not at all* (1) to *Very much* (5)	How strongly did you experience joy during this activity? *Not at all* (1) to *Very much* (5)
Trait measures*		
Control	I generally feel in control of learning situations in my studies. *Not true at all* (1) to *Completely true* (5)	–
Value	Understanding the content in my studies is personally of great importance to me. *Not true at all* (1) to *Completely true* (5)	–
Enjoyment	In learning situations in my studies, I typically experience enjoyment. *Not true at all* (1) to *Completely true* (5)	–

*State measures in Study 1 and Study 2 and trait measures in Study 1were assessed with single items.

In the following, we will present exemplary analyses on (1) intraindividual fluctuation of the constructs, (2) interindividual differences in intraindividual fluctuation, (3) comparisons of relations on the inter- and intraindividual level, (4) decomposition in intra- and interindividual variance, (5) comparisons of means and correlations between aggregated state and corresponding trait measures, and (6) a comparison of relations among aggregated state constructs and relations among trait constructs.

### Intraindividual fluctuation of constructs

**Figure 3:**
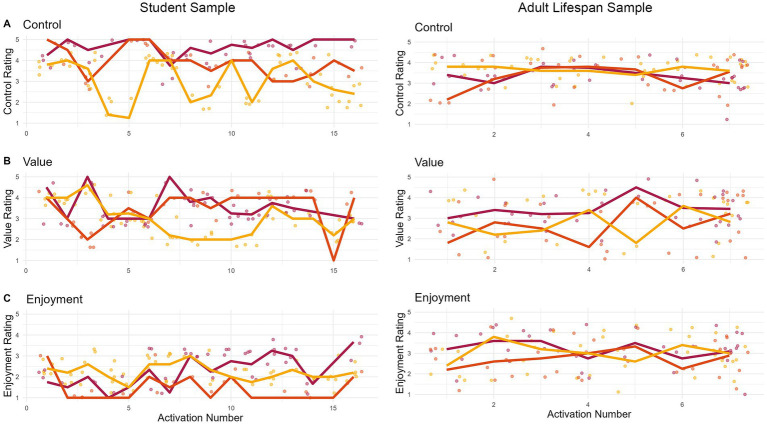
Fluctuations within participants across time. The figures display how control **(A)**, value **(B)**, and enjoyment **(C)** fluctuate within three participants across the duration of data collection. The left panel (Study 1) displays three participants (yellow, orange, and red lines) who activated the app 16 times during learning situations. As event sampling was used and participants activated the app individually, the distance between activation sessions might differ between participants. The right panel displays three randomly selected participants and their responses across seven days of assessment. For all panels, the jittered points depict single assessments nested within the respective clustering units: learning situations (Study 1) and mental situations across days (Study 2). The lines connect the situational/daily averages of the respective individuals.

The data collected with the *ESM-Quest* app allows for investigating the intraindividual fluctuation of constructs. Figure 3 displays such fluctuations in the three constructs of control, value, and enjoyment within three selected participants across time. Each color indicates one participant. Points indicate single assessments nested within learning situations (left panel, Study 1) or days (right panel, Study 2). The x-axis depicts the numbered learning situations and days, respectively. Figure 3 illustrates that there are pronounced intraindividual (= within-person) fluctuations in constructs over time. For example, in the left panel, we can see that the mean level of control experienced in learning situations changes considerably across time for participant 1 (displayed in yellow). Though less pronounced, we also observe changes in average experienced control, value, and enjoyment per day over the week of data collection in the adult lifespan sample (right panel). Here, participant 1 (displayed in yellow) displays little to no fluctuation in control, but considerable fluctuation in value, which illustrates that patterns of intraindividual fluctuation may depend on the construct in question. Using experience sampling allows us to study such intraindividual fluctuations and compare them across constructs or individuals. Research questions could aim to explain intraindividual fluctuations, for example by using contextual or dispositional aspects as predictors, or investigate temporary aspects, such as increases or decreases occurring naturally or linked to interventions.

### Interindividual differences in intraindividual fluctuation

**Figure 4:**
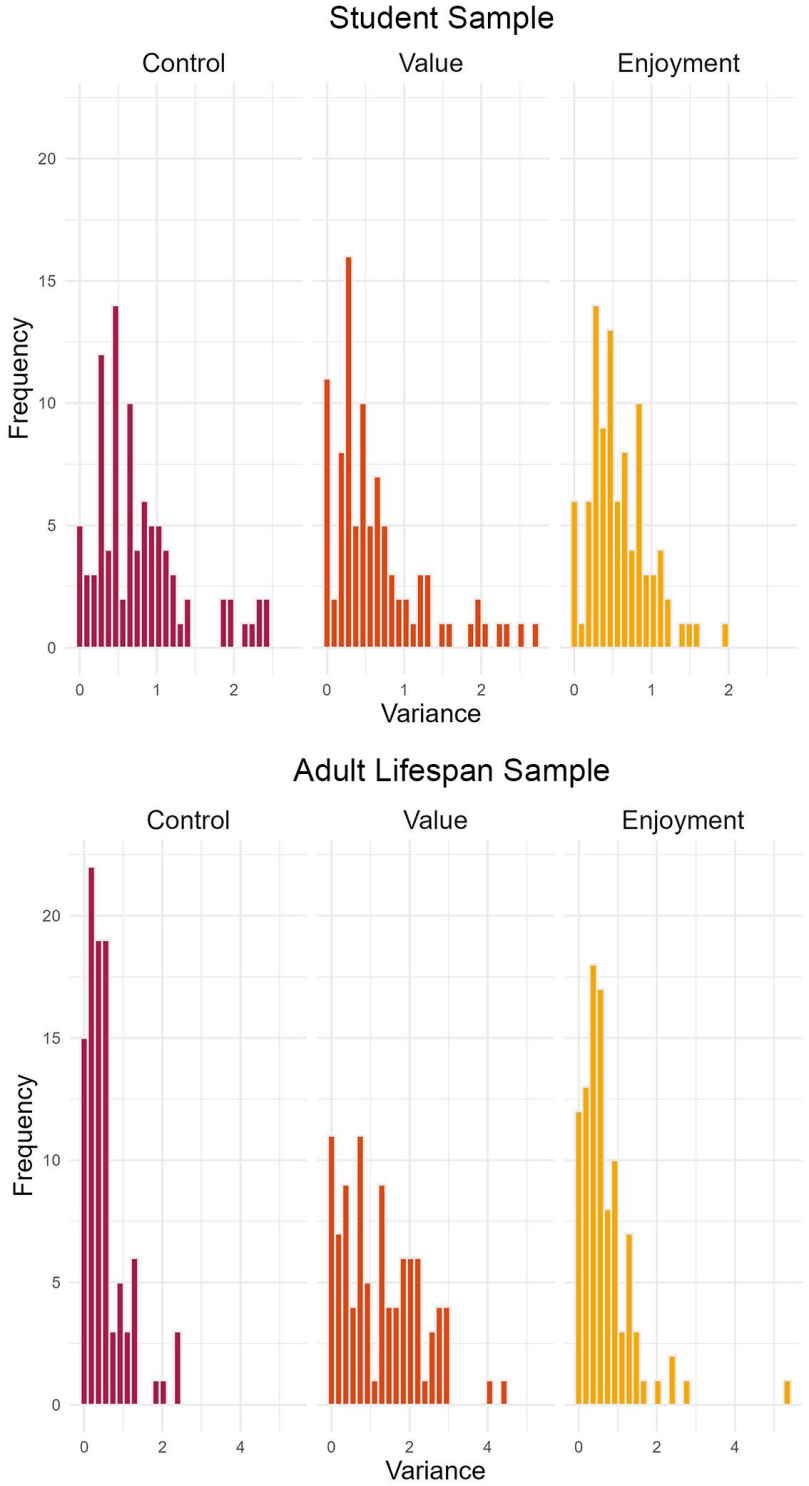
Distribution of individuals’ variance of state responses. The figure illustrates the range of intraindividual variance for the variables control, value, and enjoyment across participants, for the student sample of Study 1 (upper panel) and the adult lifespan sample of Study 2 (lower panel). For example, in Study 1 (upper panel), five participants experienced no variance in control over time (i.e., their control ratings were on the same level of intensity across all measurement points).

The data collected with the *ESM-Quest* app allows us to compare intraindividual variation across individuals. Such interindividual differences in the variance of constructs within persons are shown in Figure 4. The histograms display the amount of variance in state assessments of control, value, and enjoyment on the x-axis by individual. The y-axis indicates the number of individuals who display the respective amount of variance in their state ratings of control, value, and enjoyment. Overall, variances mostly accumulate between values of 0 and 1, indicating zero to moderate variation for most individuals. However, some individuals experience much higher variation than others (i.e., their experiences of control, value, or enjoyment are much more variable over time). The right panel (adult lifespan sample) also indicates smaller amounts of intraindividual variance in control (= fluctuates less strongly) than value (which is also illustrated by Figure 4). Research questions could aim to explain why some individuals show stronger fluctuations of constructs than other individuals. For example, individual differences such as older age or lower excitability may come with smaller fluctuations (e.g., Röcke et al., 2009). Also, fluctuations themselves may serve as predictors. Higher fluctuations and variability could be both an indicator of adaptability and resilience or, in turn, maladaptation and vulnerability depending on the circumstances and construct in question (for an overview see Mac Donald and Stawski, 2014).

### Comparing intra- and interindividual relations

The data collected with the *ESM-Quest* app allows for the analysis of intra, as well as interindividual relations (see Table 2). Intraindividual relations refer to relations between variables on the level of state assessments, which can capture temporal fluctuations. For example, if individuals perceived higher control during learning (Study 1) or performing a mental activity (Study 2) than they normally would, they also perceived relatively higher value (*r* = 0.19/0.14 for Study 1/2). Interindividual relations refer to relations between variables on the level of aggregated states across assessments. Results as outlined in Table 2 show, for example, that individuals who perceive higher control on average, also report higher value on average, in both samples (*r* = 0.29/0.49 for Study 1/2). These examples illustrate that inter- and intraindividual relations can deviate from each other. In this case, relations between control and value were weak on the intraindividual level (= situational/state assessment; Level 1), but moderate on the interindividual level (= aggregated state values; Level 2). As the majority of research and literature focuses on interindividual relations, innovative research questions could ask whether established associations on the interindividual level can also be found on the intraindividual level (see Voelkle et al., 2014; Goetz et al., 2016b; Murayama et al., 2017). That is, whether traits and dispositions are merely associated (i.e., personal tendencies to feel in control and to attribute value to cognitive situations), or whether the relations are more interwoven in a given situation.

**Table 2:** Pearson correlations for variables at the intra- and interindividual level.

	Student sample (Study 1)			Adult lifespan sample (Study 2)		
	1.	2.	3.	1.	2.	3.
1. State control	–	0.29**	0.18	–	0.49***	0.67***
2. State value	0.19***	–	0.31**	0.14***	–	0.13
3. State enjoyment	0.35***	0.26***	–	0.25***	−0.10**	–

Lower triangular = intraindividual/state variables (Study 1: *N* = 1,951; Study 2: *N* = 729); Upper triangular = interindividual/aggregates state variables (Study 1: *N* = 97, Study 2: *N* = 106); listwise deletion.

**p < 0.01.

***p < 0.001.

### Decomposition of intra- and interindividual variance

**Figure 5:**
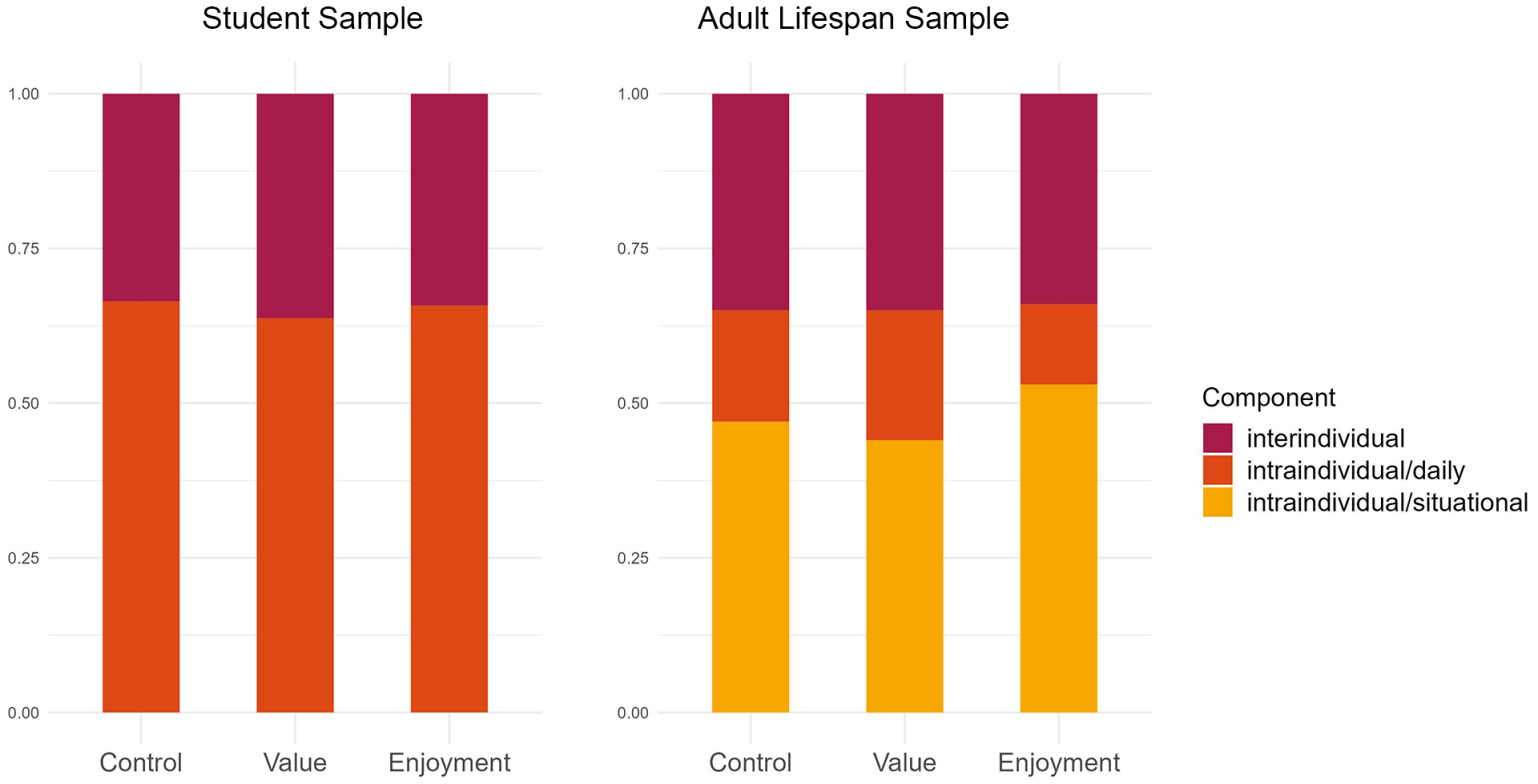
Variance decomposition in inter- and intraindividual variance components. The student sample (Study 1) is displayed in the left panel, the adult lifespan sample (Study 2) is displayed in the right panel. For the adult lifespan sample, intraindividual variance is displayed separately for daily variance (nested in individuals) and situational variance (nested in both individuals and days).

With the availability of state-data from multiple participants it is possible to inspect whether the variance of a construct is primarily driven by differences within individuals, between individuals or between other clusters (= levels) included in the data. A method to analyze how much variance is attributed to the different levels is variance decomposition. Figure 5 (left panel) depicts the variance decomposition for the Study 1 constructs, where we differentiated between intraindividual variance (= Level 1) and interindividual variance (= Level 2). It becomes evident that the larger proportion of overall variance is driven by variation within individuals.

Figure 5 (right panel) depicts the variance decomposition for Study 2, where we built a three-level structure of intraindividual variance on the situational level (= Level 1), the day level (= Level 2), as well as the interindividual level (= Level 3). Variance can be found on all three levels, with the lowest proportion on the day level and the highest on the situational (= intraindividual) level.

Importantly, the lowest level of variance also contains residual variance (i.e., variance because of assessment errors and unreliable and manifest assessment of constructs). Research questions can address which level determines the variation in a variable of interest – the situation, the person, or both – and whether a multilevel structure is indeed necessary to consider. For example, there could only be a negligible amount of variance either on the intraindividual level or the interindividual level.

### Comparing means and correlations of aggregated states with corresponding traits

Many studies have used aggregated state assessments as a proxy for the corresponding trait construct (see Conner and Barrett, 2012). However, the assumption that an aggregation of momentary experiences (i.e., states) reflects an individual’s disposition toward a certain experience (i.e., trait) is not a given. The experience sampling design in Study 1 allows us to further explore the question of aggregated state vs. trait, as we utilized items with parallel wording for trait and state measures of control, value, and enjoyment.

The trait versions of the items explicitly asked participants for an overall judgment of the control, value, and enjoyment they *usually* experience in learning situations, while state items asked for an immediate judgment of one’s current experience. These two modes of assessment allow us to not only compare trait and singular state assessments but also to compare trait assessments with the aggregated states (= individual means of state assessments, calculated by aggregating Level 1 assessments to Level 2 means).

**Figure 6:**
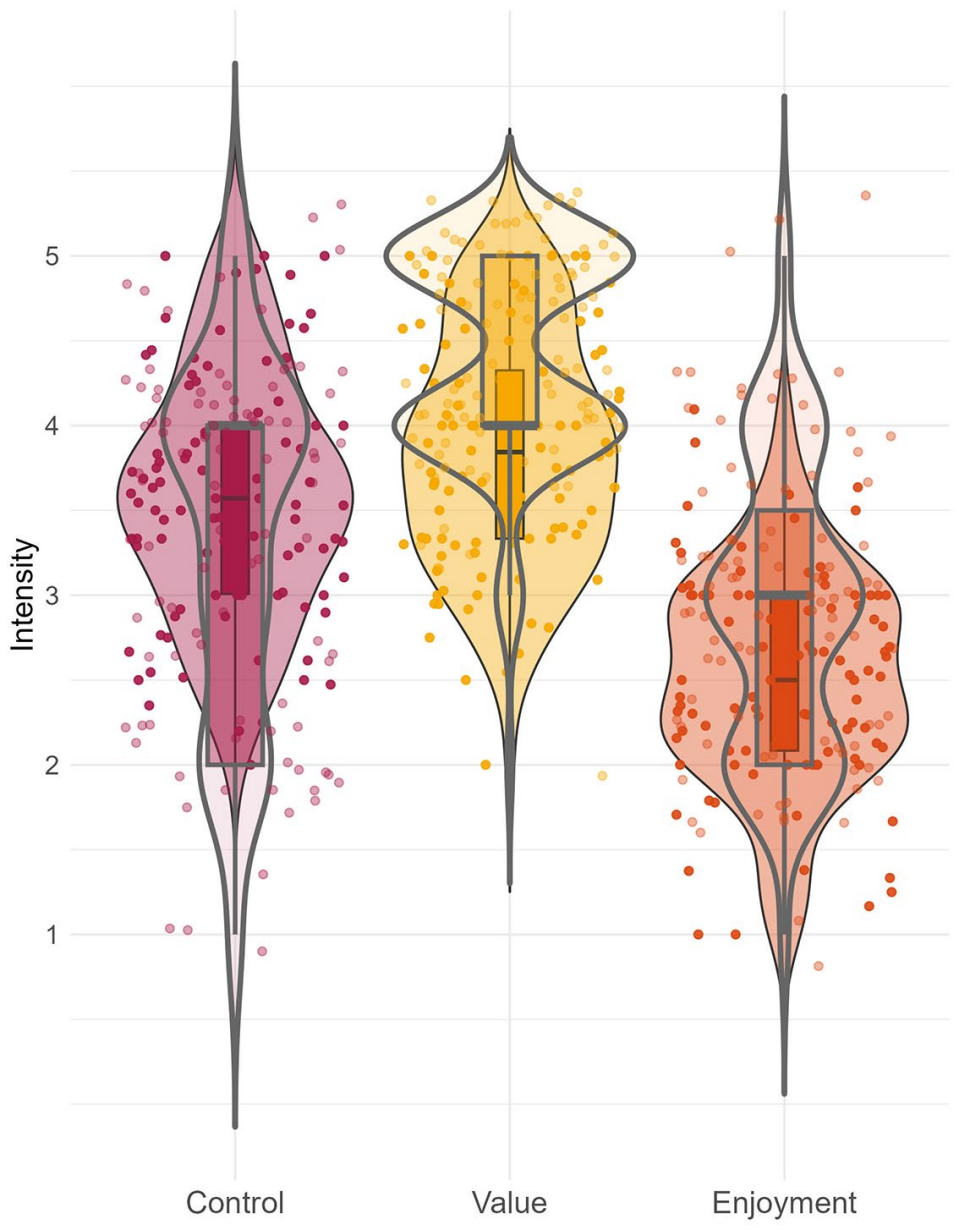
Distribution of aggregated state (background, in color) and trait (front, gray lines) variables (Study 1). Aggregated state variables are displayed in color, trait variables are displayed in gray and transparent points.

Firstly, we can compare mean values of aggregated states with mean values of traits and examine them for correspondence. As shown in Table 3, mean values of trait control are significantly lower than aggregated state means, and mean values of trait value and trait enjoyment are significantly higher than the corresponding aggregated state means. The differences in the distributions are illustrated in Figure 6, which shows the violin and boxplots of aggregated state values in color and violin plots of trait values in gray.

**Table 3:** Descriptive statistics of trait and state emotions (Study 1).

Variable	Trait assessment	Aggregated states		Wilcoxon test	
	*M*	*SD*	*M*	*SD*	*p*
Control	3.32	1.04	3.58	0.71	0.027
Value	4.41	0.64	3.83	0.69	< 0.001
Enjoyment	2.93	0.87	2.51	0.63	< 0.001

*N* = 95.

Secondly, we can analyze the relation between aggregated states and corresponding trait measures and again examine them for correspondence. Prior research has shown that relations between aggregated states and traits differ depending on the construct of analysis (see, for example, Rauthmann et al., 2019). As shown in Table 4, we find correlations of moderate effect size (*r* = 0.34 to.42, *p* < 0.001) between aggregated states and their corresponding trait measures. Using experience sampling, research questions can investigate differences in mean values and correlations between aggregated states and traits of different concepts, and thereby investigate trait–state homomorphy (i.e., whether trait and state items measure the same constructs) for different psychological constructs (Rauthmann et al., 2019).

**Table 4:** Pearson correlations for variables at the aggregated state level with corresponding trait assessments.

	Trait control	Trait value	Trait enjoyment
Agg. state control	0.42***		
Agg. state value		0.34***	
Agg. state enjoyment			0.36***

*N* = 95–97; pairwise deletion.

***p < 0.001.

### Comparing relations among aggregated states and relations among trait variables

If trait constructs represent aggregated states of those constructs, one could assume that relations among trait variables are in line with relations between corresponding variables on the level of aggregated state assessments. Using items with parallel wording for trait and state measures in Study 1 allowed us to compare these relations using the different types of measurement. For variables in the aggregated state as shown in Table 5, perceived control relates positively to perceived value (*r* = 0.22; *p* = 0.03). Interestingly, this relationship is not in line with the respective trait-level constructs (*r* = 0.07; *p* = 0.48; see Table 6). There is no significant correlation between trait control and trait value. Discrepancies like these can easily be uncovered using a combination of state and trait assessments via ESM. Research questions might address whether known associations are dependent on the measurement of the construct (e.g., as a trait or aggregated state). Further, one could investigate whether differences in associations hold when controlling for concepts which are known to influence trait rather than state responses (e.g., gender; Goetz et al., 2013).

**Table 5:** Pearson correlations for variables at the aggregated state level (Study 1).

	1.	2.	3.
1. Agg. state control	–		
2. Agg. state value	0.22*	–	
3. Agg. state enjoyment	0.17	0.30**	–

*N* = 97; no missing values; Agg. = aggregated.

*p < 0.05.

**p < 0.01.

**Table 6:** Pearson correlations for variables at the trait level (Study 1).

	1.	2.	3.
1. Trait control	–		
2. Trait value	0.07	–	
3. Trait enjoyment	0.12	0.40***	–

*N* = 95; pairwise deletion.

***p < 0.001.

## General discussion

We have provided examples of how to utilize experience sampling data, as assessed with *ESM-Quest*, in relation to specific research questions. The illustrations demonstrate that ESM data enables a wide range of analyses pertaining to inter- and intraindividual data, addressing various research inquiries, such as the analysis of (1) intraindividual fluctuations of constructs, (2) the investigation of interindividual differences in intraindividual fluctuations, (3) comparisons of relations on the inter- and intraindividual level, (4) decomposition in intra- and interindividual variance, (5) comparisons of means and correlations between aggregated state and corresponding trait measures, and (6) a comparison of correlations among aggregated state constructs and correlations among trait constructs.

However, it is important to note that those analyses are just some examples of how to use ESM data. In addition to the aforementioned examples, several other analyses are possible. For instance, in ESM research, single items are typically employed for assessments due to time constraints and to ensure data validity by avoiding lengthy questionnaires that may not be consistently completed (Gogol et al., 2014). Consequently, an inquiry arises regarding the aspects of a construct that these single ESM items primarily reflect. This could be investigated by incorporating a multi-item scale in one ESM assessment and analyzing the relationship of the single item with this scale. For example, in emotion research, a multi-item scale could evaluate various components of an emotion, such as affective, cognitive, motivational, and physiological aspects (Goetz et al., 2023). Through such analyses, it becomes possible to determine which component a single item of this emotion predominantly represents. These types of analyses extend beyond emotions and can be applied to multifaceted psychological constructs. In essence, ESM opens up numerous research avenues that were previously inaccessible with traditional trait questionnaires.

Furthermore, above and beyond our examples, other ways of analyzing ESM data might be used, such as multi-level analyses and time series analyses (Hamilton, 2020). Especially, time series analyses might be very helpful for analyzing causal relations within individuals. Including ESM assessments in longitudinal designs could be highly insightful in understanding how causal relationships unfold over time (e.g., measurement burst designs; e.g., Sliwinski, 2008).

In sum, through our examples on how to analyze ESM data and by providing hints regarding other possible analyses, we aim to motivate researchers to conduct ESM studies. This method has the potential to yield highly valuable data, allowing for numerous analyses within and between individuals. In combination with longitudinal designs, it can provide insight into how relationships unfold on different timescales, such as within days, months, or years. The experience-sampling app *ESM-Quest*, as introduced in this paper, offers a rather easy technical solution for implementing ESM studies.

### Future directions in ESM research on psychological variables

Future directions in ESM research on psychological variables might be to combine experience sampling with other types of assessments. Even self-report, as used in ESM, might generally be a good choice for the assessment of psychological variables, however, self-report variables have limitations in that they are restricted to accessible processes and bear the possibility of self-report biases. Therefore, it can be useful to complement self-report with other methods, such as physiological indicators and observation of facial expression (while considering that these methods have their own limitations in terms of reduced sensitivity and/or specificity; see, e.g., Harley et al., 2015).

A highly important area of future ESM research lies in its application within the realm of adaptive systems, which have experienced a notable surge in significance in recent years. For example, adaptive technical learning systems have become increasingly prominent as they allow for a more individualized type of learning. For instance, computerized adaptive testing (CAT; e.g., Wainer, 2000; Asseburg and Frey, 2013) can be useful in reducing situations of non-optimal challenge during tests, where individuals may be either over- or underchallenged. In CAT, items are selected individually based on the test takers’ previous responses. Therefore, if a wrong answer is given, an easier item will be presented next, and vice versa. In learning situations as well, the difficulty of the material can be adjusted based on students’ current competence level on the topic. This testing strategy is, for example, expected to significantly reduce boredom, which is a common result of being over- or underchallenged (Goetz et al., 2023). However, adaptive systems can go beyond considering just competence level and can also take psychological variables such as metacognition, motivation, and emotions into account. For example, specific content areas on a particular difficulty level within a domain can be selected based on these psychological variables (e.g., more abstract or concrete material). For example, if a system recognizes a decrease in a student’s enjoyment while working on highly abstract material, it might react by presenting more concrete materials with the aim of rekindling enjoyment. Real-time assessments are essential for the use of such adaptive technical learning systems, with the quality of adaptive systems heavily relying on accurately assessing an individual’s current state. In this regard, the ESM is an incredibly valuable tool. As demonstrated in this paper, emotions, for instance, can fluctuate significantly within students or adults engaging in mental activities. Thus, real-time reactions of adaptive systems are warranted based on ongoing assessments of these fluctuations. It is important to note that, currently, self-report as used in ESM is the only valid way to assess the affective component of emotional experiences in real-time (Pekrun et al., 2023). Likewise, other psychological variables like metacognitive and motivational constructs can only be assessed to a limited extent beyond self-report.

With respect to the outlined future directions, but also beyond those lines of research, the presented app, *ESM-Quest*, can be a highly valuable tool for data collection. First of all, as it allows event-based random sampling, it is possible to assess randomized data within given situations. For example, academic emotions, being highly domain-specific in nature (Goetz et al., 2007), can be assessed in one specific domain (e.g., math classes) by activating the app, for instance, at the beginning of a class, and then conducting a number of randomized assessments during the class. In other words, *ESM-Quest* allows a focus on specific domains within the academic context and beyond (e.g., doing sports, eating, shopping), enabling analyses regarding these domains and potential differences across them.

## Data availability statement

The raw data supporting the conclusions of this article will be made available by the authors, without undue reservation.

## Ethics statement

Ethical approval was not required for the studies involving humans because all participants were older than 18 years, and none of the assessment questions posed any potential harm to the participants. The studies were conducted in accordance with the local legislation and institutional requirements. The participants provided their written informed consent to participate in this study.

## Author contributions

TG: Conceptualization, Investigation, Writing – original draft. WS: Data curation, Investigation, Methodology, Software, Visualization, Writing – original draft, Project administration. EG: Conceptualization, Formal analysis, Investigation, Methodology, Writing – original draft, Project administration. LS: Conceptualization, Formal analysis, Investigation, Methodology, Writing – original draft, Project administration. CR: Conceptualization, Formal analysis, Investigation, Methodology, Project administration, Writing – original draft. FR: Conceptualization, Formal analysis, Investigation, Methodology, Project administration, Writing – original draft. JD: Conceptualization, Investigation, Methodology, Project administration, Writing – original draft. EB: Conceptualization, Investigation, Methodology, Project administration, Writing – original draft. JN: Conceptualization, Investigation, Writing – original draft.

## Footnotes

^1^https://esm.univie.ac.at
